# Infection of *Brachypodium distachyon* by Formae Speciales of *Puccinia graminis*: Early Infection Events and Host-Pathogen Incompatibility

**DOI:** 10.1371/journal.pone.0056857

**Published:** 2013-02-18

**Authors:** Melania Figueroa, Stephen Alderman, David F. Garvin, William F. Pfender

**Affiliations:** 1 Forage Seed and Cereal Research Unit, Agricultural Research Service, U.S. Department of Agriculture, Corvallis, Oregon, United States of America; 2 Plant Science Research Unit and Department of Agronomy and Plant Genetics, Agricultural Research Service, U.S. Department of Agriculture, University of Minnesota. St. Paul, Minnesota, United States of America; 3 Department of Botany and Plant Pathology, Oregon State University, Corvallis, Oregon, United States of America; China Agricultural University, China

## Abstract

*Puccinia graminis* causes stem rust, a serious disease of cereals and forage grasses. Important formae speciales of *P. graminis* and their typical hosts are *P. graminis* f. sp. *tritici* (*Pg-tr*) in wheat and barley, *P. graminis* f. sp. *lolii* (*Pg-lo*) in perennial ryegrass and tall fescue, and *P. graminis* f. sp. *phlei-pratensis* (*Pg-pp*) in timothy grass. *Brachypodium distachyon* is an emerging genetic model to study fungal disease resistance in cereals and temperate grasses. We characterized the *P. graminis*-*Brachypodium* pathosystem to evaluate its potential for investigating incompatibility and non-host resistance to *P. graminis*. Inoculation of eight *Brachypodium* inbred lines with *Pg-tr*, *Pg-lo* or *Pg-pp* resulted in sporulating lesions later accompanied by necrosis. Histological analysis of early infection events in one *Brachypodium* inbred line (Bd1-1) indicated that *Pg-lo* and *Pg-pp* were markedly more efficient than *Pg-tr* at establishing a biotrophic interaction. Formation of appressoria was completed (60–70% of germinated spores) by 12 h post-inoculation (hpi) under dark and wet conditions, and after 4 h of subsequent light exposure fungal penetration structures (penetration peg, substomatal vesicle and primary infection hyphae) had developed. *Brachypodium* Bd1-1 exhibited pre-haustorial resistance to *Pg-tr*, i.e. infection usually stopped at appressorial formation. By 68 hpi, only 0.3% and 0.7% of the *Pg-tr* urediniospores developed haustoria and colonies, respectively. In contrast, development of advanced infection structures by *Pg-lo* and *Pg-p*p was significantly more common; however, *Brachypodium* displayed post-haustorial resistance to these isolates. By 68 hpi the percentage of urediniospores that only develop a haustorium mother cell or haustorium in *Pg-lo* and *Pg-pp* reached 8% and 5%, respectively. The formation of colonies reached 14% and 13%, respectively. We conclude that *Brachypodium* is an apt grass model to study the molecular and genetic components of incompatiblity and non-host resistance to *P. graminis*.

## Introduction

*Puccinia graminis* Pers.: Pers., causal agent of stem rust, is an obligate biotroph that belongs to the rust fungi (Pucciniales, Basidiomycota) [1], a group that includes some of the most diverse and economically important fungal pathogens of crops [2]. The variability in host range and morphology among members of the species *P. graminis* has created a challenge in establishing a consistent taxonomic nomenclature for subspecific populations [3], [4], [5]. There are two coexisting systems for the classification of *P. graminis*. One system, based on spore morphology, divides the species into two taxonomic subspecies (subsp.), *graminis* and *graminicola* [3], [6]. *P. graminis* subsp. *graminis* infects primarily cereal crops and closely related genera, whereas *P. graminis* subsp. *graminicola* infects mostly non-cereal grasses. The other system classifies *P. graminis* according to host range, and separates the species into different formae speciales (f. sp.) [7]. In either system, rust genotypes can be designated as physiological races based on the virulence of the pathogen genotype to a specific set of genotypes within the host species [8]. For convenience, this paper uses the formae speciales designation, but we note that *P. graminis* f. sp. *tritici*, the causal agent of wheat stem rust, is included in *Puccinia graminis* subsp. *graminis*, whereas *P. graminis* f. sp. *lolii* and *P. graminis* f. sp. *phlei-pratensis* are included in *P. graminis* subsp. *graminicola* [3], [7], [9], [10].

*P. graminis* f. sp. *tritici* (*Pg-tr*) is one of the most destructive pathogens of common wheat (*Triticum aestivum* L.), durum wheat (*Triticum turgidum* L. var. *durum*) and barley (*Hordeum vulgare* L.) because it weakens the stem of the plant while disrupting nutrient uptake and evapotranspiration control, leading to shriveled grain [11]. Other formae speciales of *P. graminis* are responsible for causing stem rust in grasses and affecting the production and quality of forage and seed. *Puccinia graminis* f. sp. *lolii* (*Pg-lo*) is a major pathogen of perennial ryegrass (*Lolium perenne* L.) and tall fescue (*Festuca arundinacea* Schreb) [9], [12], two important cool-season forage and turf type grasses [13], [14], whereas *P. graminis* f.sp. *phlei-pratensis* (*Pg-pp*) infects timothy grass (*Phleum pratense* L.), another perennial grass used as a forage crop [11]. *Puccinia graminis* has a complex life cycle that produces five types of spores [11]. The single-celled dikaryotic urediniospores, which are generated on and can re-infect the gramineous host, play a crucial role in stem rust outbreaks as they permit a continuous cycle of the disease [11]. Contact between the urediniospore and water on the leaf surface during darkness triggers growth of a germ tube that elongates perpendicular to the long axis of the host epidermal cells [15]. The germ tube usually extends until it finds a stoma, and proceeds to form an appressorium [11]. In wheat, the germination of urediniospores of *Pg-tr* occurs by 2 hours post-inoculation (hpi) under optimum conditions [16]. The formation of appressoria can be detected at 6 hpi [17] and maximum appressorium development is reached by 12 hpi [16]. Subsequent to appressorium formation, light and photosynthesis-associated O_2_ reduction stimulate the growth of a penetration peg and a substomatal vesicle in the mesophyll space [18]. Substomatal vesicles form within 1.5 h after light exposure [19] and a primary infection hypha emerges from the vesicle, and eventually differentiates a haustorial mother cell. Each haustorial mother cell generates a peg that penetrates the wall of a mesophyll cell to form a haustorium [20]. Haustoria facilitate nutrient uptake by the fungus and secretion of effectors that suppress host defense responses, a process necessary for the establishment of the fungal colony [15], [21]. After formation of the first haustorium, the body of the fungus branches to generate secondary infection hyphae [11]. In the wheat-*Pg-tr* pathosystem, the formation of first haustoria can be observed between 16 and 20 hpi [16] and emergence of secondary infection hyphae occurs between 24 and 36 hpi under experimental conditions in which a light period follows a 12 h dark period [17].

Aside from the gramineous host, the infection cycle of *P. graminis* involves an alternate host (*Berberis* or *Mahonia*), and its eradication is a commonly used method to control the pathogen [11]. The application of fungicides is another method to control stem rust outbreaks [11]; however, this approach has environmental and economic costs. For years, discovery and incorporation of resistance genes into wheat cultivars has provided a solution to mitigate and prevent stem rust epidemics [11], but the emergence of a highly virulent race of *Pg-tr*, TTKSK (also known as Ug99) has re-awakened serious crop safety concerns [22], [23]. Breeding for stem rust resistance in forage grasses has been delayed due to their relatively recent domestication and their outbreeding mating systems. Thus, in both cereals and grasses, genetic resistance to *P. graminis* remains a high priority in crop improvement.

In the past several years there has been an increasing interest in non-host resistance (NHR), as it is considered a resource for durable resistance that could be transferred to crops [24], [25]. NHR is defined as the resistance that is common across all genotypes of a plant species and prevents the establishment of any genetic variants (i.e., formae speciales, races, isolates) of a given would-be pathogen [25], [26]. A would-be pathogen that is unable to circumvent NHR is known as an unadapted pathogen. The interaction between an unadapted pathogen and a non-host plant leads to either the absence of symptoms or the elicitation of necrosis (cell death) associated with hypersensitive response (HR). It is well-established that NHR consists of constitutive and/or inducible pre- and post-penetration responses [27], [28]. Constitutive defenses, considered the first layer of NHR, are provided by preformed physical and chemical barriers [24]. If the unadapted pathogen overcomes these defenses, inducible plant defenses are activated. NHR can be mediated by PAMP (pathogen-associated molecular patterns)-triggered immunity (PTI) [29] and/or the defense reactions evoked during effector-triggered immunity (ETI), which are typically associated with host resistance [30], [31], [32]. Thus, the term NHR is applied to a state that may include a range of responses with overlapping signaling pathways. In addition to this conceptual complexity, the study of the molecular and genetic mechanisms underlying NHR has been difficult due to the polygenic and multilayered nature of this type of resistance [28], [33]. Further adding to the difficulty of studying NHR, a classical genetic investigation would require interspecific crosses between host and non-host plant species, which are typically unattainable or produce progenies that can exhibit sterility and abnormal segregation [34].

Biotrophic pathogens are prime organisms to investigate various types of resistance, including NHR, as they differentiate sophisticated infection structures and cause relatively minimal damage to the host cell during the nutrient-transfer process [35]. Ayliffe et al. [36] have directed their efforts to dissecting the molecular mechanisms of NHR to *P. graminis* by using natural and mutagenized variants of rice. These studies are valuable to understand NHR in gramineous plants, but the applicability of rice as a model for temperate cereals and forage grasses is not straightforward [37]. *Brachypodium distachyon* (hereafter referred to as *Brachypodium*) is a wild grass that exhibits traits amenable to use as a research model for the study of temperate forage grasses and cereals [37], [38]. Given its phylogenetic position within the Pooideae subfamily [37] and the strong syntenic relationships with barley [39] and wheat [40], *Brachypodium* is an ideal plant to provide genomic insight into members of Triticeae (cereals) and Poeae (forage grasses) tribes. *Brachypodium* can be infected by various fungal pathogens of cereals and grasses [41], [42] and displays variation in natural resistance to *P. striiformis* [43] and *P. triticina* [44]. Natural variation in resistance to *P*. *graminis* also has been reported [45]. Here, we compare the ability of *Pg-tr*, *Pg-lo*, and *Pg-pp* to infect *Brachypodium*. We also characterize, at the phenotypic and histological level, the range of the interactions that occur in *P. graminis*-*Brachypodium* pathosystems. Our findings indicate that *Brachypodium* is a suitable plant to investigate mechanisms of stem rust resistance, and holds promise as a genetic resource for breeding programs.

## Materials and Methods

### *Brachypodium distachyon* inbred lines

Seed of *Brachypodium* inbred lines Bd1-1, Bd2-3, Bd3-1, Bd18-1, Bd21, Bd21-3, Bd29-1, and Bd30-1 were employed in this study [46]. All inbred lines, except Bd30-1, derived from accessions in the USDA National Plant Germplasm System (http://www.ars-grin.gov/npgs). Inbred line Bd30-1 derived from an accession collected in Spain by Dr. A. Manzaneda.

### Natural hosts and origin

Wheat (*Triticum aestivum* L.) cultivar (cv.) McNair 701, used as a natural host for *P. graminis* f. sp. *tritici* (*Pg-tr*), was obtained from Dr. Carl Griffey (Virginia Tech Agronomy Farm, Blacksburg, VA). Perennial ryegrass (*Lolium perenne* L.) and timothy grass (*Phleum pratense* L.) were used as natural hosts for *P. graminis* f. sp. *lolii* (*Pg-lo*) and *P. graminis* f. sp. *phlei-pratensis* (*Pg-pp*), respectively. Perennial ryegrass cv. Jet and timothy grass cv. Toro were obtained from Pennington Seed Co. (Lebanon, OR) and USDA-ARS, Washington State University-Regional Plant Introduction Station (Pullman, WA), respectively. Vegetative clones (ramets) of perennial ryegrass and timothy grass were generated by dividing plants to five tillers and repotting.

### Plant materials and growth conditions

Plants were grown in a greenhouse maintained at 19°C and 13°C during the day and night, respectively. Natural light was supplemented with artificial lighting (high-pressure sodium lamps, 430 W; Philips Lighting Somerset, NJ) to extend daylength to 14 h. A single seed per pot for each plant was grown in soil (Sunshine Growing Mix; Sun Gro Horticulture Inc., Bellevue, WA) in 3.8×21-cm cylindrical cones (“Cone-tainer”, Stuewe & Sons Inc., Corvallis, OR) and fertilized every 2 weeks with a pH-adjusted NPK solution (6 mg N, 5.4 mg P_2_O_5_ and 6 mg K_2_O in 4 L water). To promote synchronous seedling germination of *Brachypodium*, seeds were stratified at 4°C with 8 h of fluorescent light for a minimum of 5 days, and moved to the greenhouse afterwards. Wheat, perennial ryegrass and timothy grass were irrigated from below by placing plant racks in ∼6 cm of water for 2 days per week. *Brachypodium* seedlings were watered every 2 days or as needed. All plants except *Brachypodium* were periodically trimmed to a height of ∼8 cm. Wheat plants were inoculated at 7 days of age, perennial ryegrass and timothy grass were inoculated 2 weeks after vegetative cloning, and *Brachypodium* seedlings were inoculated 3 weeks after emergence.

### Origin, maintenance and preparation of stem rust inoculum

Single pustule isolate cultures of *Pg-lo* isolate 106 [13] and *Pg-tr* isolate 1101 (race GFCDC) were obtained from inoculum collected in agricultural fields (Oregon, USA) as previously described [13]. Urediniospores collected from a single isolated pustule were put through 2–3 cycles of inoculation and cleanup on fresh susceptible plants in a growth chamber or small designated greenhouse before increasing inoculum. The single pustule *Pg-pp* isolate was developed from material collected in Minnesota (USA) and stored at the USDA-ARS Cereal Disease Laboratory. Stocks of inoculum for each pathogen were produced as previously described [13]. Urediniospores were collected from each of the isolates, and desiccated overnight at 30% relative humidity over a KOH solution [13]. Urediniospores were stored in gelatin capsules at −60°C and heat shocked at 43°C for 1.5 min before use [47]. Two types of experiments were conducted: 1) to compare disease severity and symptom development among fungal isolate/host combinations, different inbred lines of *Brachypodium* were inoculated separately with *Pg-tr*, *Pg-lo* or *Pg-pp*; and 2) to perform a histological comparative analysis on early events in the infection process, *Brachypodium* line Bd1-1 was inoculated with *Pg-tr*, *Pg-lo* or *Pg-pp*.

### Inoculations, data collection and analysis to assess disease severity

A suspension of 6 mg/ml of urediniospores of each *P. graminis* isolate was prepared in a light-weight mineral oil (Isopar M®; Exxon Mobile Chemical Co., Houston, TX) and sprayed onto plants at a rate of 0.025 ml/plant, which resulted in an inoculum density of ∼160 spores/cm^2^ as determined by counting the number of spores deposited on a microscope glass slide placed among plants before inoculation. Spore germination percentage was calculated by placing a 50 µl aliquot of the spore suspension onto a 1% water agar plate, and the spores were allowed to germinate for 24 h in the dark at RT. After inoculation, plants were allowed to dry for at least 1 h at room temperature (RT, 28°C±2) and placed into a dark dew chamber that supplied intermittent mist (15 min/2 h) for 12 h at 20°C±4. This incubation was followed by 4 h of fluorescent light as previously described [48]. Next, plants were moved to a growth chamber (day/night 24°C/17°C, 16-h light period) where symptom development was monitored for 12 days. The experiment was conducted twice, as a completely-randomized design (plants were randomized before inoculation), with 5 or 4 replicate plants (1 per pot) per treatment in trials 1 and 2, respectively. The natural host corresponding to each of the fungal isolates was included in each trial.

The severity of disease (number of sporulating lesions) on each plant was recorded at 12 days post-inoculation (dpi) to compare fungal isolate/host combinations. To adjust for possible differences among inoculum doses of the different fungi, all severity values were adjusted to a common effective dose calculated from the counts of spores per cm^2^ (on the glass slides) and percent germination (from agar plates). Before statistical analysis, data were transformed to square root values to normalize the variance. Analysis of variance was conducted using SigmaPlot (SPSS Inc., Chicago), as a 3-way ANOVA with *Brachypodium* line, fungus isolate, and trial as the main effects. To document symptom development, leaves collected at various times after inoculation were imaged with a scanner (Epson, Long Beach, CA), and also photographed using an Olympus E-10 digital camera adapted to an Olympus SZH dissecting scope at a 20× magnification (Olympus America, Center Valley, PA).

### Inoculations and sample collection for histological analysis

Individual leaves of *Brachypodium* line Bd1-1 were inoculated with pure urediniospores, i.e., spores not suspended in any carrier. To reduce the likelihood of clusters of spores, an aliquot of 4 mg urediniospores was first passed through a standard 150 µm sieve (Newark Wire Cloth Co., Newark, NJ) by means of a 10/0 round artist brush (Princeton Art & Brush Co., Princeton, NJ). Spores were collected on a microscope glass slide and a cotton swab was pressed onto the slide to pick up spores that were then applied to by lightly rolling the swab on the surface of the tertiary leaf of 3-week old Bd1-1 seedlings. One freshly-loaded swab was used for each inoculated leaf. The inoculum density was ∼200 spores/cm^2^, as estimated by counting the number of spores at the point of inoculation on a sample leaf (4 microscope transects on one leaf). For each inoculation, an additional cotton swab with spores was lightly pressed on a 1% water agar plate, and the spores were allowed to germinate for 24 h in the dark at RT in order to estimate spore germination (%). After inoculation the plants were lightly misted with ddH_2_O and placed in the dew chamber under conditions described above. The experiment was repeated 3 times to produce 3 trials for analysis. Each trial consisted of 4 replicates (pots) per fungus per time point: 12, 18, 32, 42 and 68 hours post inoculation (hpi). Inoculated leaves were collected and sectioned into 1 cm-pieces before fixing the tissue. In each experiment, 2 additional control plants inoculated with each isolate were maintained intact for 12 days in the greenhouse to monitor full symptom development and assure that the experimental conditions had favored successful infection.

Two types of observation were made to characterize early infection events. Whole-mounts of inoculated leaf tissue were examined microscopically to quantify the proportion of germinated spores that had reached various morphological development stages at each time point (hpi). Alternatively, sectioned tissue was examined to record in finer resolution the appearance of the fungal isolate/host interaction at the specific sampling times. For both types of observations, samples were stained with Uvitex 2B fluorescent dye (Polysciences, Inc. Warrington, PA) prepared in 0.1 M Tris-HCl buffer (pH 5.8). Samples were examined using a Zeiss microscope with UV illumination and filter set 2 (G 365 excitation, FT 395 beam splitter, and PL 420 emission), which allowed visualization of the light blue fluorescence displayed by the fungus. Photomicrographs were taken with a Moticam 2500 digital camera and Motic Images Plus v.2 digital software.

### Whole-mount staining and data collection

Two replicates (inoculated leaves) from each time point (hpi) of the infection trial were fixed in 95% ethanol. Fixing and clearing of the tissue required at least 5 days at RT. Leaf sections were then rinsed in 50% ethanol for 10 min, treated with 0.05 N NaOH for 5 min and rinsed in ddH_2_O for 5 min. Afterwards, the leaves were soaked in 0.1 M Tris-HCl buffer (pH 5.8), stained in 0.1% Uvitex 2B for 2 min, and washed four times in ddH_2_O (10 min each) before being mounted in 25% aqueous glycerol on a glass slide. Observations of each fungal isolate x time point combination were taken immediately after staining and involved the classification and counting of the number of infection structures present in the first 100 urediniospores (fungal units) observed. The fungal units were classified as: 1) non-germinated spores, 2) germinated spores, 3) germinated spores with differentiated appressoria, 4) germinated spores that display one or all of the following structures: penetration peg, substomatal vesicle and primary infection hyphae, 5) germinated spores that exhibited a haustorium mother cell or haustoria, and 6) germinated spores that established a colony. For each fungal isolate x time point in each experiment, 3 randomly selected 1 cm-leaf sections were taken from each vial and placed on a microscope slide to collect data. Data were acquired by scanning the first leaf section (left to right) at 20× and 40× magnification until 100 fungal units were classified. If the first leaf section did not contain sufficient spores, the second and third section was examined until 100 fungal units were observed.

The number of fungal units at each stage of development within a sample was converted to a percentage, using total number of observable units (germinated urediniospores at any stage of development) as the denominator. These data were transformed to arcsin square root before conducting the analysis to compare the treatments (fungi) within each sampling time. Data were analyzed by 2-way ANOVA, with fungus and trial as the main effects. No statistical comparisons were made among or between sampling times.

### Preparation of longitudinal leaf sections and staining

Sections from two inoculated leaves from each time point (hpi) of the infection trial were fixed in FAA solution [containing per 100 ml: 50 ml 95% ethanol, 5 ml glacial acetic acid, 10 ml formalin (37% formaldehyde solution), 35 ml dH_2_O], vacuum infiltrated and stored at RT. After a minimum of 24 h, samples were rinsed in ethanol, dehydrated and infiltrated with paraffin using tert-butyl alcohol as the intermediate solvent, as detailed by Ruzin [49]. Sections were cut at 10 µm using a Reichert rotary microtome and carbide knife. Sections were applied to slides coated with an aminopropyl-triethoxysilane slide coating adhesive (Electron Microscopy Sciences, Fort Washington, PA). Sections on slides were dewaxed in xylene for 3 min, hydrated through an ethanol series (100%, 95%, 70%, 50%, and 30%, 3 min per step), stained in 1% aq safranin for 50 sec, rinsed in water to remove excess safranin, stained in 0.025% Uvitex 2B for 10 sec, rinsed in water for 2 min and mounted in glycerol. Safranin stain was used to provide contrast for visualizing plant structures, especially lignified plant tissue, which is stained dark red by this dye.

## Results

### Variation for stem rust resistance in *Brachypodium*

**Figure 1:**
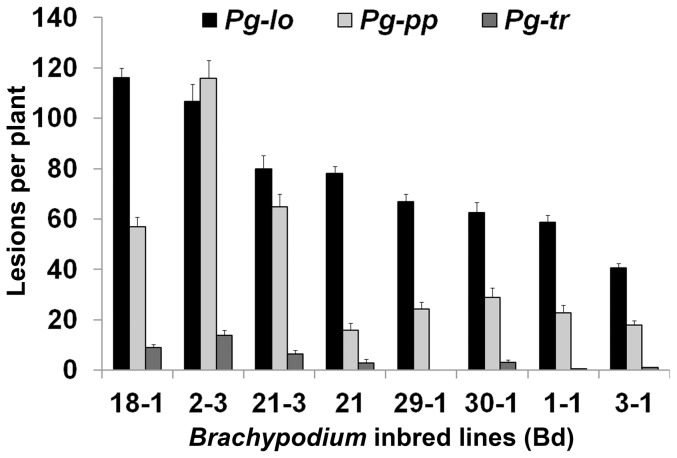
Pattern of stem rust severities on *Brachypodium* inbred lines inoculated with urediniospores of *P. graminis* f. sp. *lolii* (*Pg-lo*), *P. graminis* f. sp. *phlei-pratensis* (*Pg-pp*) or *P. graminis* f. sp. *tritici* (*Pg-tr*). Data were collected at 12 days post-inoculation. Bar height represents the mean of total number of sporulating lesions per plant across two trials. Error bars indicate standard error of the mean for each treatment, pooled across trials. Results from statistical analysis are shown in Table 1.

We examined eight *Brachypodium* inbred lines for their response to *P. graminis* f. sp. *tritici* (*Pg-tr*), *P. graminis* f. sp. *lolii* (*Pg-lo*) or *P. graminis* f. sp. *phlei-pratensis* (*Pg-pp*). Data on number of sporulating lesions per plant in the different fungal isolate/host combinations demonstrated clear patterns for disease severity (Figure 1). Statistical analysis showed significant main effects for fungal isolate, *Brachypodium* line and trial. Interactions for fungal isolate x host, and pathogen x trial were statistically significant (*P*<0.01), whereas host x trial and the three-way interaction were not statistically significant (*P*>0.2). *Pg-tr* caused significantly less disease (*P*<0.001, analysis not shown) than either *Pg-lo* or *Pg-pp* on each *Brachypodium* line in both trials of the experiment, in some lines forming no visible lesions (Figure 1; Figure S1). *Pg-lo* caused significantly (*P*<0.01) more disease than *Pg-pp* in both experiments for most *Brachypodium* lines; for lines Bd18-1 and Bd2-3 this difference was not statistically significant. Mean separation results among *Brachypodium* lines for each fungal isolate and both experiments are shown in Table 1. To summarize, *Brachypodium* lines Bd2-3, Bd18-1, and Bd21-3 had significantly more disease per standardized inoculum dose than lines Bd1-1 or Bd3-1 for all fungal isolates and both trials, except *Pg-pp* in trial 1. The other *Brachypodium* lines (Bd21, Bd29-1 and Bd30-1) showed intermediate levels of disease, variously similar to the high-disease or low-disease *Brachypodium* lines among the different fungal isolates and trials (Table 1).

**Table 1:** Disease severity of *Brachypodium* inbred lines challenged with *P. graminis* f. sp. *lolii* (*Pg-lo*), *P. graminis* f. sp. *phlei-pratensis* (*Pg-pp*) and *P. graminis* f. sp. *tritici* (*Pg-tr*)*.

	*Pg-lo*		*Pg-pp*		*Pg-tr*	
Bd line§	Trial† 1	Trial† 2	Trial† 1	Trial† 2	Trial† 1	Trial† 2
Bd18-1	9.4, a	11.8, a	6.6, b	8.3, b	1.1, a	3.9, a
Bd2-3	9.0, ab	11.2, a	9.2, a	12.0, a	3.3, a	3.8, a
Bd21-3	7.7, abc	9.6, a	6.7, ab	9.1, bc	2.0, a	2.3, a
Bd21	6.5, bc	10.6, ab	3.3, c	4.3, d	1.6, a	0.9, b
Bd29-1	6.4, cd	9.6, ab	3.5, c	5.8, cd	0.0, b	0.0, b
Bd30-1	5.9, cd	9.1, ab	4.2, b	6.1, c	1.6, a	1.4, a
Bd1-1	6.1, cd	8.4, b	4.8, b	4.4, d	0.6, b	0.0, b
Bd3-1	4.3, d	7.8, bc	3.2, c	5.0, d	0.6, b	0.4, b

§ *Brachypodium distachyon* (Bd) inbred lines.

† Trial number.

* Numbers within columns represent means of √ (sporulating lesions per plant). Numbers within a column followed by the same letter do not differ (*P* = 0.01) according to Tukey's test.

**Figure 2:**
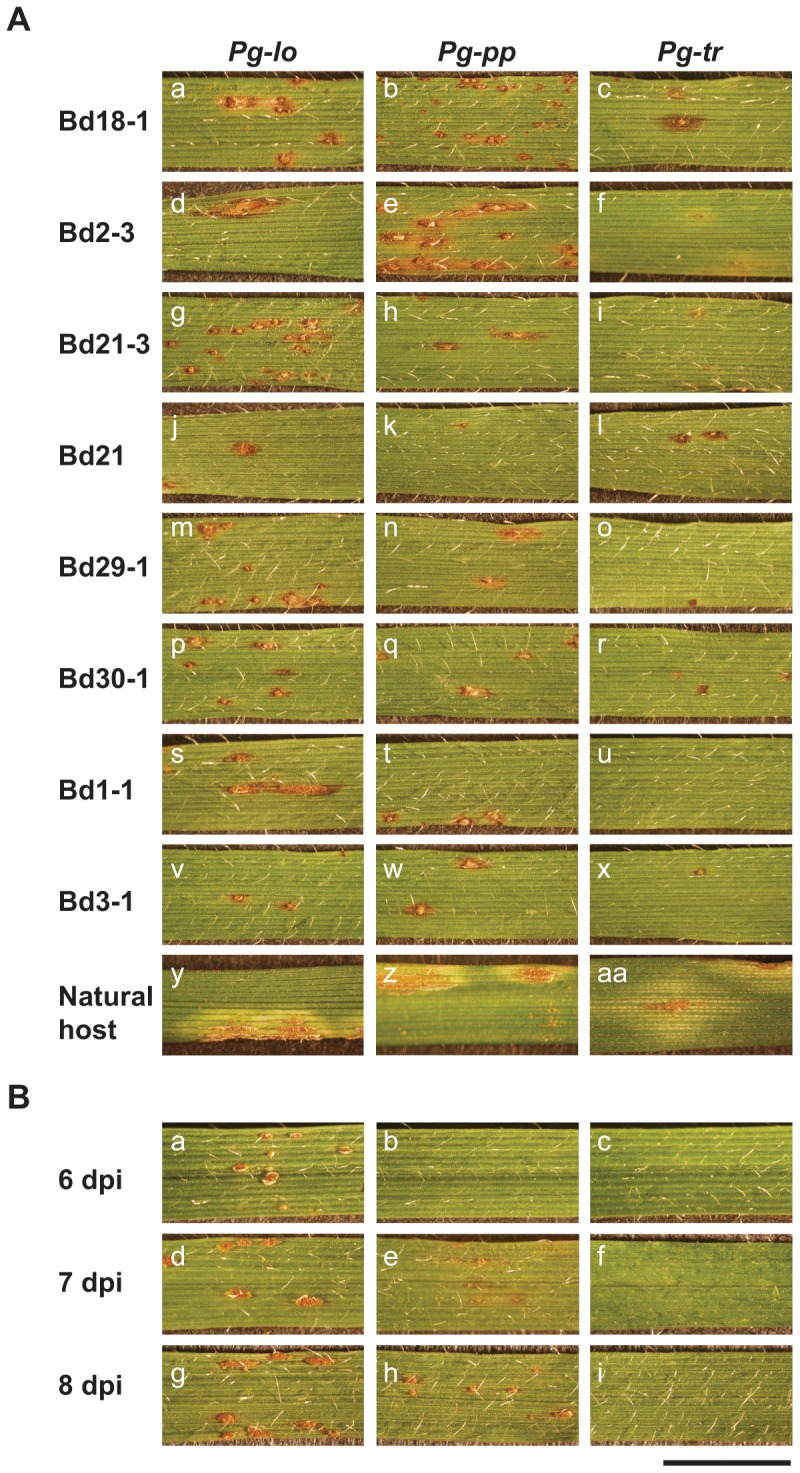
Variation of symptom phenotypes induced by the different stem rust formae speciales. (**A**) Close-up image of the sporulating lesions developed in the different *Brachypodium* inbred lines and natural hosts inoculated with fungal isolates *P. graminis* f. sp. *tritici* (*Pg-tr*), *P. graminis* f. sp. *lolii* (*Pg-lo*), and *P. graminis* f. sp. *phlei-pratensis* (*Pg-pp*). (**B**) Time of symptom appearance in *Brachypodium* inbred line Bd1-1 according to fungal isolates. Scale bar indicates 5 cm.

Besides differences in number of lesions produced in the various fungal isolate/host combinations, the three fungal isolates produced a wide range of reactions that manifested in different disease phenotypes (lesion size, presence and extent of chlorotic and/or necrotic halo surrounding the lesion). When present, the lesions that developed on *Brachypodium* were considerably smaller than the sporulating lesions that occur in the natural hosts (Figure 2A, compare panels a through x to panels y through aa). Based on phenotypic comparisons between the symptoms that developed in the *Brachypodium* lines and natural host plants, none of the *Brachypodium* lines can be classified as fully susceptible to any of the fungal isolates. Nonetheless, *Pg-lo* and *Pg-pp* did produce sporulating lesions that persisted for up to 12 days on many of the *Brachypodium* lines.

The sporulating lesions produced on *Brachypodium* lines inoculated with *Pg-lo* were usually small (Figure 2A, g, j, m, p, s, v); Figure S1, left panel), except in lines Bd1-1 (Figure 2A, s) and Bd2-3 (Figure 2A, d), and to some extend Bd21 (Figure 2A, j) and Bd18-1 (Figure 2A, a), which often presented larger lesions with substantial fungal sporulation. In addition to the necrotic symptoms present around lesions on each *Brachypodium* inbred line, *Pg-lo* induced chlorosis associated with lesions in inbred lines Bd1-1 (Figure 2A, s), Bd2-3 (Figure 2A, d), Bd18-1 (Figure 2A, a; Figure S1), and occasionally in Bd29-1 (Figure 2A, t) and Bd3-1 (Figure 2A, v). All *Brachypodium* lines inoculated with *Pg-pp* developed lesions that were surrounded by necrotic tissue (Figure 2A, b, e, h, k, n, q, t, w; Figure S1, middle panel). Whereas most of the inbred lines inoculated with *Pg-pp* typically displayed small sporulating lesions, lines Bd1-1 (Figure 2A, t) and Bd2-3 (Figure 2A, e), showed some larger lesions. Additionally, lesions in these two lines were accompanied by chlorosis.

When challenged with *Pg-tr*, all *Brachypodium* inbred lines showed either absence of symptoms, or development of dark flecks or poorly-sporulating, small lesions (Figure 2A, c, f, i, l, o, r, u and x, Figure S1, right panel). In most of the *Brachypodium* lines the *Pg-tr* lesions were surrounded by necrotic tissue. However, in lines Bd2-3 (Figure 2A, f), and especially Bd3-1 (Figure 2A, x) and Bd21-3 (Figure 2A, i) the extent of necrosis was very limited, almost null. The lesions in line Bd2-3 were accompanied by spreading chlorosis (Figure 2A, d). In contrast, lines Bd18-1 (Figure 2A, c) and Bd21 (Figure 2A, l) showed extensive necrotic halos around the lesions. In general, the majority of the lesions that resulted from the different fungal isolate/*Brachypodium* interactions had limited sporulation. As time passed the extent of necrosis around each lesion increased, which led to collapsing and shrinking of the sporulating lesions.

### Time course of symptom development

One striking and general difference among the stem rust isolates was the number of days necessary for lesions to become visible in the *Brachypodium* lines. By 6 dpi, Bd1-1 inoculated *Pg-lo* showed symptom development (Figure 2B, a), with no signs of necrosis. In contrast, by 6 dpi, line Bd1-1 inoculated with either *Pg-pp* (Figure 2B, b) or *Pg-tr* (Figure 2B) lacked symptoms. By 7 dpi, *Pg-lo* lesions on Bd1-1 displayed neither chlorosis nor necrosis and continued to enlarge (Figure 2B, d). At this time, *Pg-pp* lesions on Bd1-1 plants became erumpent with initial signs of necrosis and chlorosis (Figure 2B, e). By 8 dpi, some of the *Pg-lo* lesions present on Bd1-1 began to show minor signs of necrosis (Figure 2B, g), while *Pg-pp* showed a more evident combination of necrotic and chlorotic halos around the lesions (Figure 2B, h). The pustules developed by *Pg-pp* appear to grow at a slower rate than the *Pg-lo* pustules. Symptoms in Bd1-1 in response to *Pg-tr* were rare and not detected until 8 dpi or later (Figure 2A, f, i). Collapse and shrinking of the pustules developed by each of the three fungal isolates began between 13–15 dpi as the necrosis and/or chlorosis expanded, especially in the case of *Pg-lo* and *Pg-pp* (data not shown).

### Histological characterization of early events of *P. graminis* infection in *Brachypodium*

**Figure 3:**
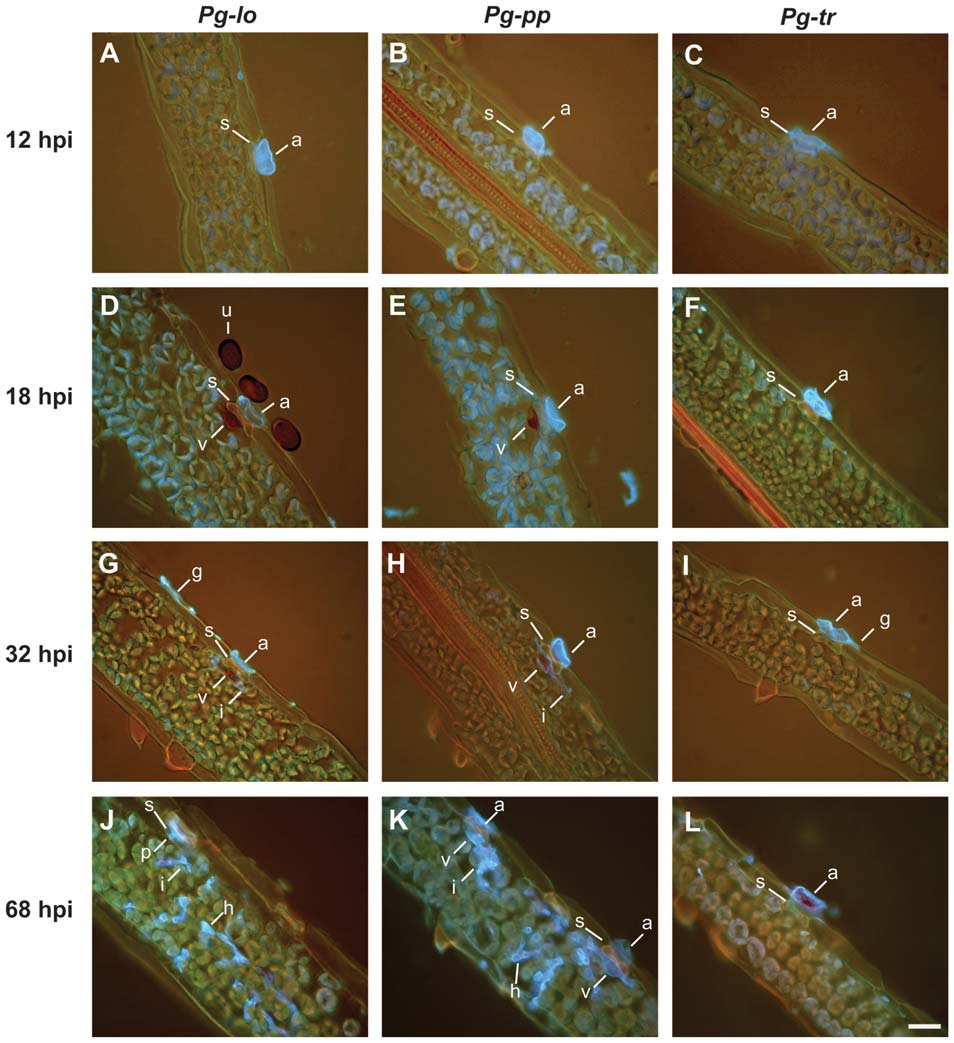
Early events in the infection of *Brachypodium* inbred line Bd1-1 by *Puccinia graminis* formae speciales. Fluorescence micrographs of longitudinal leaf sections show the fungal penetration sites and formation of infection structures according to time in hours post-inoculation (left side). The isolates *P. graminis* f. sp. *tritici* (*Pg-tr*), *P. graminis* f. sp. *lolii* (*Pg-lo*), and *P. graminis* f. sp. *phlei-pratensis* (*Pg-pp*) used for inoculation are indicated at the top of the figure. Fungal tissue was stained with Uvitex 2B (chitin specific) which exhibits blue fluorescence under UV-light. Structures are labeled as follows: u, urediniospore; g, germ tube; a, appressorium; s, leaf stoma; i, primary infection hyphae; v, substomatal vesicle; h, haustorium. Panels D, E, G and H show the formation of callose papillae below fungal penetration sites. Scale bar in panel L indicates 20 µm.

**Figure 4:**
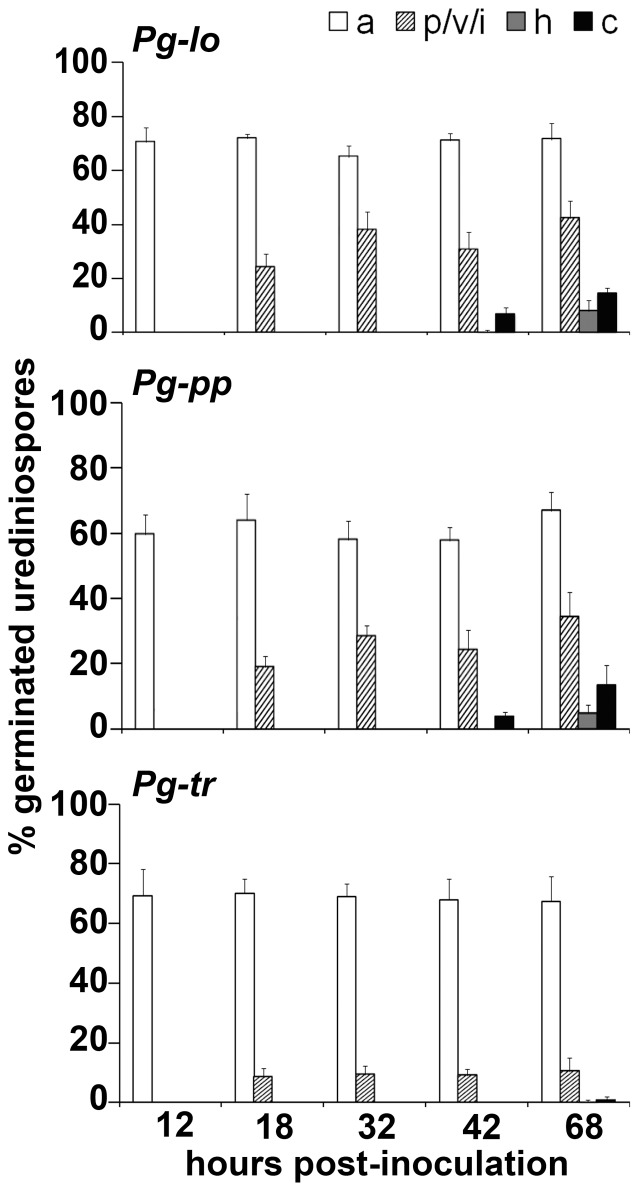
Time course of the differentiation of infection structures by *Puccinia graminis* formae speciales in *Brachypodium* inbred line Bd1-1. Top panel shows *P. graminis* f. sp. *lolii* (*Pg-lo*), middle panel shows *P. graminis* f. sp. *phlei-pratensis* (*Pg-pp*), and bottom panel shows *P. graminis* f. sp. *tritici* (*Pg-tr*). The percentage of germinated urediniospores that formed an appressorium (a) at the end of the germ tube is indicated by solid white bars. The percentage of germinated urediniospores that reached the formation of either a penetration peg (p), a substomatal vesicle (v) and/or primary infection hyphae (i) are indicated by the white and grey striped bars. The percentage of germinated urediniospores that reached the stage of haustorium mother cell or primary haustorium (h) is shown by the gray solid bars. The percentage of germinated urediniospores that established a colony (c) is shown by black solid bars. Bar heights indicate the averages of total percentages, and error bars represent standard deviation.

Given the differences in disease severity, lesion character and development time among the three fungal isolates (Figure 1, Figure 2A, Figure S1), we sought to characterize the early infection events that occurred during these fungal-plant interactions. A staining technique combining Uvitex 2B and safranin was developed to visualize the fungal and plant structures and detect lignin containing-cell wall appositions at the fungal penetration sites. Urediniospores from all three *P. graminis* formae speciales had a similar percentage of *in planta* germination: *Pg-tr*, 94%; *Pg-lo*, 96%; *Pg-pp*, 95% (*P* = 0.54, ns). After completion of a 12 h-period of moisture in darkness, all three fungal isolates had formed appressoria (Figure 3, panels A, B and C). Total percentage of germinated urediniospores forming a mature appressorium was equal or greater than 60% for all three fungi: 70% for *Pg-lo*, 60% for *Pg-pp* and 69% for *Pg-tr* (Figure 4). At this sampling time, and averaged across all sampling times, appressorium formation by *Pg-pp* was significantly (*P*<0.05) less than for the other two fungi, which did not differ from one another. Inoculation of Bd1-1 with either *Pg-lo* or *Pg-pp* resulted in a biotrophic interaction. After 4 h of light exposure (18 hpi), penetration structures (penetration peg, substomatal vesicle and/or primary infection hyphae) were differentiated for 24% and 19% of the total germinated urediniospores of *Pg-lo* and *Pg-pp*, respectively (Figure 4, top and middle panels). At all sampling times, there was no statistically significant difference between *Pg-lo* and *Pg-pp* in proportion of units forming penetration structures, but *Pg-tr* formed significantly fewer (*P*<0.01). Most of the penetration structures consisted of a substomatal vesicle that was often encapsulated by cell wall appositions or papillae (Figure 3, panels D and E). By 32 hpi, the percentage of germinated urediniospores of *Pg-lo* and *Pg-pp* with penetration structures increased to 38% and 28%, respectively (Figure 4, top and middle panels). In some instances at 32 hpi, the fungal hyphae escaped encapsulation (Figure 3, panels G and H, notice i). By 42 hpi (Figure 4, top panel), 30% of total germinated urediniospores of *Pg-lo* had produced penetration structures but no advanced fungal invasive structures, 0.3% were at the stage of a differentiated haustorium mother cell or mature haustorium, and 7% had formed a colony. After 68 hpi (Figure 4, top panel), 42% of total germinated urediniospores of *Pg-lo* remained at the stage of penetration structures, 8% had developed only as far as a haustorium mother cell or a mature primary haustorium, and 14% had established a colony that was widely spread among the mesophyll cells (Figure 3, panel J). In the case of *Pg-pp*, by 42 hpi (Figure 4, middle panel), 24% of the total germinated spores exhibited penetration structures and no other fungal invasive structures, and 4% of the total germinated urediniospores had formed a colony. By 68 hpi (Figure 4, middle panel), 34% of the total germinated spores of *Pg-pp* presented penetration structures but no other invasive structures, 5% germinated spores were at the stage of a haustorium mother cell or a mature primary haustorium, and 13% of the total germinated urediniospores had attained colony formation (Figure 3, panel K). *Pg-lo* and *Pg-pp* did not differ statistically (*P*>0.05) in formation of haustoria or colonies at the 42 or 68 hpi sampling times.

In contrast to *Pg-lo* and *Pg-pp*, only 8.5% of the total germinated urediniospores of *Pg-tr* had formed some penetration structure by 18 hpi, (Figure 4, bottom panel) and this percentage remained constant throughout the rest of the time-course samples for this fungus. At 68 hpi, 0.3% of the germinated urediniospores had formed a visible haustorium and only 0.7% had successfully formed a colony (Figure 4, bottom panel). That is, most of the infection sites for *Pg-tr* did not exhibit post-appressorial fungal growth (Figure 3, panels F, I and L), and the percentage of germinated urediniospores that formed haustoria or colonies was significantly (*P*<0.05) less for *Pg-tr* than for the other two fungi. Thus, during the early infection events (up to 68 hpi), there were essentially no morphological differences between *Pg-lo* and *Pg-pp*, whereas *Pg-tr* showed markedly less development to a successful biotrophic interaction.

## Discussion

In the last decade, there has been an major effort to develop *Brachypodium* as a plant research model [50]. This includes the acquisition of genome sequences of various inbred lines, such as Bd21 [51] and Bd1-1 (Vogel et al. unpublished data), as well as the construction of genetic linkage [52], [53] and physical maps [50], [54]. Here, we evaluated disease development to assess the utility of the *P. graminis*-*Brachypodium* pathosystem to investigate the molecular and genetic basis of stem rust resistance. Evaluation of disease severity and lesion phenotypes in *Brachypodium* diploid inbred lines inoculated with *P. graminis* f. sp. *tritici* (*Pg-tr*), *P. graminis* f. sp. *lolii* (*Pg-lo*) or *P. graminis* f. sp. *phlei-pratensis* (*Pg-pp*) demonstrated the variation in stem rust resistance (from partially susceptible to almost immune) that occurs in *Brachypodium* (Figure S1, Figures 2 and 3). The appearance of dark flecks or necrosis around the pustules caused by all three *P. graminis* isolates (Figure 2A) is evocative of hypersensitive response (HR) and non-host resistance (NHR) [27]. Thus, the range in resistance to *P. graminis* isolates among different *Brachypodium* genotypes could be the consequence of yet undetermined NHR mechanisms and/or other specific host resistance genetic components, such as R (resistance) genes. The present study provides initial data to hypothesize the mechanisms that control host resistance to stem rust in *Brachypodium*. Future investigations are required to address the nature of these responses and identify the molecular and genetic factors that contribute to them.

Our findings revealed the different symptom-inducing capabilities of each fungal isolate (Figure 1). In general, we classify *Pg-tr* as the least virulent isolate, followed by *Pg-pp*, and finally *Pg-lo* as the most virulent isolate among the three. However, none of the fungal isolates produced a fully compatible interaction with any of the *Brachypodium* lines (Figure 2). The difference between *Pg-tr* and the other two isolates in disease severity (number of lesions) on line Bd1-1 is explained by the fact that only a very small proportion of the *Pg-tr* germinated spores succeed in differentiating penetration structures and establishing a biotrophic interaction. Preliminary data suggest that such pre-haustorial host resistance also occurs in line Bd21 when inoculated with *Pg-tr* (Pfender et al., unpublished data). In the case of *Pg-lo* and *Pg-pp* it is evident that inbred line Bd1-1 exhibits post-haustorial resistance. Although there are examples in the literature of NHR exhibited as a post-haustorial event [36], it has been proposed that non-host reactions to rust are usually pre-haustorial. Additionally, it is considered that host resistance controlled by major resistance genes typically does not manifest until the primary haustorium is established [55], [56]. If one accepts that generalization, our results suggest that line Bd1-1 displays NHR to *Pg-tr*, whereas other more specific mechanisms related to host incompatibility might dictate the resistance to *Pg-lo* and *Pg-pp.* Categorizing the interactions of Bd1-1 with *Pg-lo* or *Pg-pp* as incompatibility rather than NHR is consistent with the designation of *Brachypodium* as a host for *P. graminis* subsp. *graminicola* [3], which includes *Pg-lo* and *Pg-pp*.

As an example of NHR, the development of *Pg-tr* in rice has been examined previously [36]. Interestingly, the morphological characteristics of *Pg-tr* in rice are significantly different than those in *Brachypodium*. In rice, *Pg-tr* forms advanced infection structures (infection hyphae and haustoria) and the outcome of the interaction is the establishment of colonies that vary in size but do not develop to macroscopically-visible pustules [36]. Thus, *Brachypodium* and rice, as non-hosts to *Pg-tr*, act differently. The cellular morphological responses that take place in *Brachypodium* challenged with *Pg-lo* and *Pg-pp* resemble those reported for *Pg-tr* and rice [36], which suggests a similarity in the genetic components of both pathosystems. In response to *Pg-tr*, rice produces callose deposition at fungal penetration sites along with production of H_2_O_2_ and occasional cell death [36]. A common response of Bd1-1 to both *Pg-lo* and *Pg-pp* was the accumulation of lignin and the formation of papillae (callose deposition) at the site of fungal penetration by 18 hpi (Figure 3). Papillae formation also occurs in *Brachypodium* after being challenged with the powdery mildew pathogens *Blumeria graminis* f. spp. *hordeii*, *avenae* and *tritici* [37]. Cell wall fortification by papillae at the point of fungal penetration are considered to act as a barrier to pathogen penetration, a response typical of pathogen-host incompatibility [57]. Interestingly, *Pg-lo* and *Pg-pp* can occasionally escape encapsulation by papillae (Figure 3). This escape is reminiscent of a phenomenon known as HR trailing, which refers to the inefficacy of HR to impede the growth of the pathogen, as the pathogen outruns the plant defense response and hyphae continue to grow [31].

One typical characteristic of R-gene mediated resistance is the arrest of colony growth, often accompanied by necrosis. For example, colony growth of an incompatible isolate of *Pg-tr* in wheat containing the resistance gene *Sr6* is arrested by 60 hpi, after the onset of host cell necrosis at 24–26 hpi [16]. In the case of *Pg-tr* race Ug99, the effect of an undetermined resistance gene in the near-isogenic wheat line Thatcher-*Sr31* is shown by differences in colony size evident after 5 days [58]. The presence of necrosis and the small size of the lesions in *Brachypodium*, especially in the case of *Pg-lo* and *Pg-pp*, indicates growth arrest of the fungus. This observation suggests that there are similarities between incompatible *Pg-tr*-wheat interactions and the resistance responses of *Brachypodium* to *P. graminis*. Although direct comparisons are difficult to make due to effects of the genetic background of the host and pathogen, we contrast the fungal development of *P. graminis* observed in *Brachypodium* with that previously reported in wheat and barley. According to our histological analysis in Bd1-1 (Figure 4), the growth of *Pg-tr*, *Pg-lo* and *Pg-pp* in *Brachypodium* during the first 12 hpi (under dark, wet conditions) is comparable to that of *Pg-tr* in susceptible or resistant wheat cultivars [16]. Under comparable experimental conditions, the formation of substomatal vesicles and primary hyphae of *Pg-lo* and *Pg-pp* follow a time course similar to that reported in compatible and incompatible interactions between *Pg-tr* and wheat [16], [17], [18], [19] or barley [59], [60]. However, compared to *Pg-tr* in its natural hosts, *Pg-lo* and *Pg-pp* in *Brachypodium* display a delay in the formation of first haustoria (16 to 20 hpi in wheat and 18 hpi in barley vs. 32 hpi in *Brachypodium*) [16], [60] and emergence of secondary infection hyphae (24 to 36 hpi in wheat and barley vs. 42 hpi in *Brachypodium*) [16], [17], [60]. It is possible that slight differences in temperature of the experiments (20°C in wheat [16] vs. 24°C in *Brachypodium*) or other experimental conditions could account for observed differences in fungal development, but multiple plant defense mechanisms may play a role in delaying the growth of these stem rust isolates.

The differences in time course of symptom development and in final disease severity between *Pg-lo* and *Pg-pp* are not explained by early morphological events of infection, as both fungal isolates have a similar appearance during the first 68 hpi (Figure 4). There are several possible explanations for the difference in disease severity between *Pg-lo* and *Pg-pp*. The formation of advanced infection structures (such as haustorium mother cell, primary haustorium, secondary haustoria) is not a completely synchronized process; therefore, it is possible that a difference in the number of colonies does not manifest until after 68 hpi. We should note that the manner by which histological data were collected did not allow comparison of the absolute number of colonies/cm^2^; thus, differences between *Pg-lo* and *Pg-pp* could already have been established and were undetected. Variation in nutrient uptake and/or colony sporulation, as well as differences in the effector repertoire that these fungal isolates might harbor, could also influence disease development. Another factor may be the timing of plant responses to the pathogen and the strength (amplitude) of those responses, as they also can affect the outcome of a host-microbe interaction. The fashion in which the sporulating lesions collapse over time due to the expansion of necrosis and chlorosis suggests that *Brachypodium* is capable of mounting a defense response against *P. graminis*, although in a delayed manner. This is supported by the reduction in symptom development that is observed in later-developing leaves when some *Brachypodium* genotypes are re-inoculated with *Pg-tr* two weeks after the primary leaves had been inoculated [45].

Our findings encourage the use of the *P. graminis*-*Brachypodium* pathosystem as a tool for the research community interested in plant-pathogen interactions. Here, we provide a foundation for future studies to investigate the plant defense mechanisms and regulatory pathways that dictate stem rust disease incompatibility in non-host species. The genetic tractability of *Brachypodium* should help to unravel the genetics of stem rust resistance in this model plant. Significant progress has been made towards building a collection of recombinant inbred lines (RILs), obtained from crosses between the most and least stem rust resistant *Brachypodium* inbred lines [45]. Segregation analysis in response to *Pg-lo*, *Pg-pp* and *Pg-tr* should aid the identification of the major and minor genes responsible for stem rust resistance, as well as those regulatory networks that contribute to the outcome of *P. graminis*-*Brachypodium* interactions. Taking advantage of the available genomic resources for *Brachypodium*, a comparative transcriptome analysis of the early (pre-haustorial) responses of Bd1-1 to *Pg-lo*, *Pg-pp* and *Pg-tr* is currently underway. Characterization of gene expression will also help to gain insight into the complexity of the pathways that govern stem rust resistance.

## Supporting Information

Figure S1 **Symptom development induced by** ***P. graminis*** **f. sp.** ***lolii*** **,** ***P. graminis*** **f. sp.** ***phlei-pratensis*** **, or** ***P. graminis*** **f. sp.** ***tritici*** **on various** ***Brachypodium*** **inbred lines.** Leaves were collected 12 days post-inoculation.
(TIF)
