# Supplementary figures and images for: Infection of Brachypodium distachyon by Formae Speciales of Puccinia graminis: Early Infection Events and Host-Pathogen Incompatibility
Source: PLoS One. 2013 Feb 18;8(2):e56857. doi: 10.1371/journal.pone.0056857 (PMC3575480; doi:10.1371/journal.pone.0056857)

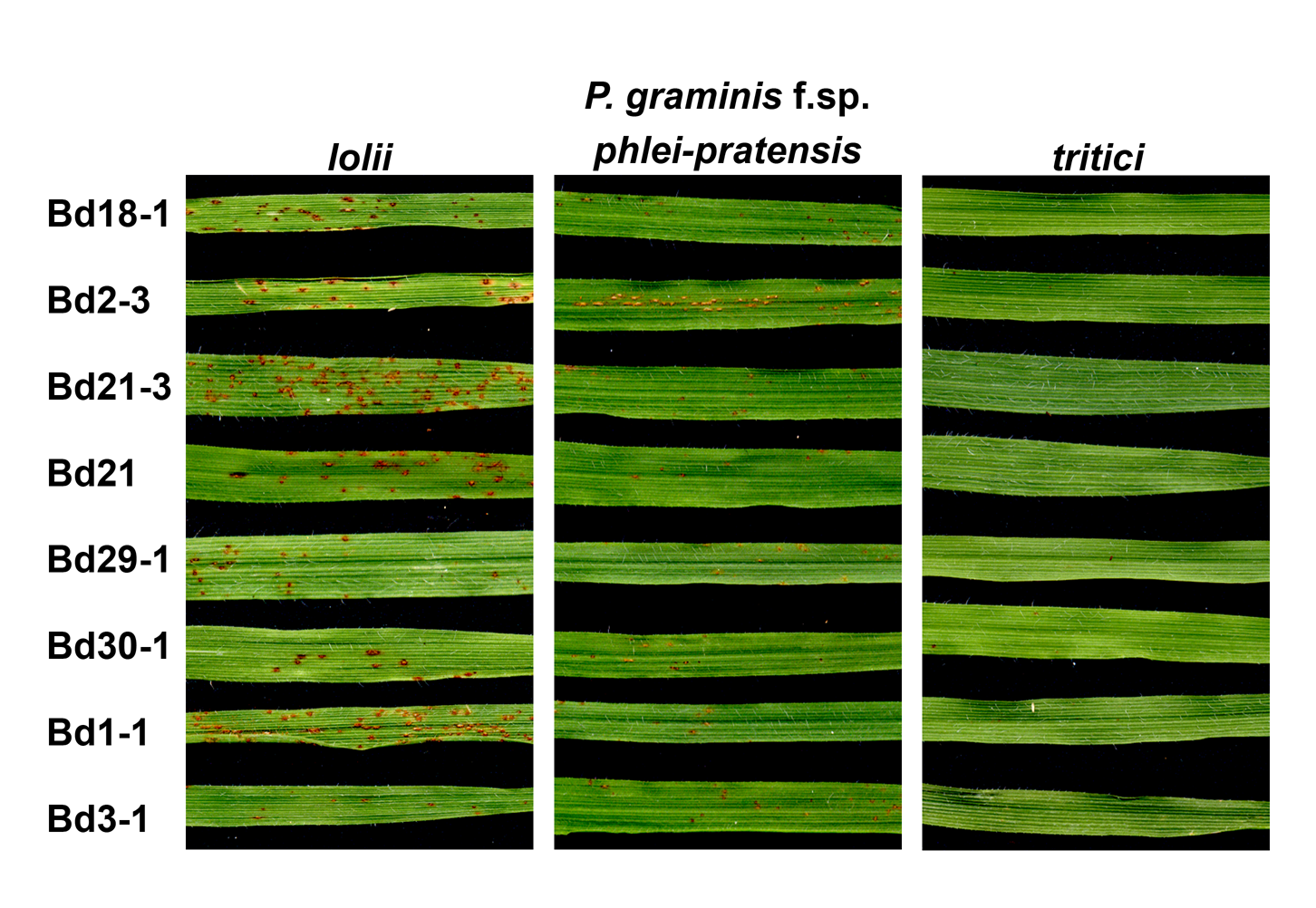

Supplement: Figure S1 — Symptom development induced by P. graminis f. sp. lolii , P. graminis f. sp. phlei-pratensis , or P. graminis f. sp. tritici on various Brachypodium inbred lines. Leaves were collected 12 days post-inoculation. (TIF) [file pone.0056857.s001.tif]
